# A deep learning latent variable model to identify children with autism through motor abnormalities

**DOI:** 10.3389/fpsyg.2023.1194760

**Published:** 2023-05-18

**Authors:** Nicola Milano, Roberta Simeoli, Angelo Rega, Davide Marocco

**Affiliations:** ^1^Department of Humanistic Studies, University of Naples Federico II, Napoli, Italy; ^2^Neapolisanit S.R.L. Rehabilitation Center, Ottaviano, Italy

**Keywords:** machine learning, autism spectrum disorder, ASD, motor abnormalities, deep learning, early detection, diagnosis

## Abstract

**Introduction:**

Autism Spectrum Disorder (ASD) is a by-birth neurodevelopmental disorder difficult to diagnose owing to the lack of clinical objective and quantitative measures. Classical diagnostic processes are time-consuming and require many specialists’ collaborative efforts to be properly accomplished. Most recent research has been conducted on automated ASD detection using advanced technologies. The proposed model automates ASD detection and provides a new quantitative method to assess ASD.

**Methods:**

The theoretical framework of our study assumes that motor abnormalities can be a potential hallmark of ASD, and Machine Learning may represent the method of choice to analyse them. In this study, a variational autoencoder, a particular type of Artificial Neural Network, is used to improve ASD detection by analysing the latent distribution description of motion features detected by a tablet-based psychometric scale.

**Results:**

The proposed ASD detection model revealed that the motion features of children with autism consistently differ from those of children with typical development.

**Discussion:**

Our results suggested that it could be possible to identify potential motion hallmarks typical for autism and support clinicians in their diagnostic process. Potentially, these measures could be used as additional indicators of disorder or suspected diagnosis.

## 1. Introduction

Autism spectrum disorder (ASD) is a neurodevelopmental disorder characterised by communication and social impairment and restricted, repetitive, and stereotyped behaviours. The aetiology of the disorder is still unknown, and it can involve both genetic and environmental factors. A high variability of manifestations notoriously characterises ASD, which makes ASD diagnostic process complicated, time-demanding and dependent on human subjectivity. Many specialists assume that autism can be classified into different types, each of which may have a different aetiology and response to treatment (Cerasuolo et al., 2022). Currently, the diagnostic process involves a series of tests that may take hours of clinical examination, and they are valid only after the child is 3 years old (e.g., Autism Diagnostic Observation Schedule, ADOS; Lord et al., 2012). These classical gold-standard tools have been widely adopted in ASD clinical practice, bringing several constraints (Volkmar et al., 2009), such as (i) the absence of explicit sensory functioning assessment, (ii) the examiner’s expertise and his subjective evaluation, (iii) the ecological validity of the assessment setting, and (iv) the time-demanding process involved. Nevertheless, early detection is necessary for early intervention, which is generally crucial for children and families, especially in neurodevelopmental disorders. For this reason, the scientific community is currently deepening the etiopathogenesis of the disorder and refining the diagnostic and assessment methods for ASD (Bertoglio and Hendren, 2009; Fombonne, 2009). Namely, researchers have begun to reconsider autistic symptomatology highlighting the potential of motion analysis (Anzulewicz et al., 2016; Simeoli et al., 2020, 2021). Although motor impairments have been widely observed in ASD, their importance as “defining symptoms” has always been underestimated. However, motor impairments in ASD significantly impact the quality of life and social development of ASD (Lai et al., 2014). They can occur very early in development (Teitelbaum et al., 1998; Brian et al., 2008) and may become more evident over the years (Fournier et al., 2010) evolving into a pervasive feature of the disorder. Several manifestations of these motor abnormalities have been detected. They can include abnormalities in walking patterns (e.g., Rinehart and McGinley, 2010; Nobile et al., 2011; Gong et al., 2020), hand movements such as reaching and grasping (e.g., Forti et al., 2011; Bäckström et al., 2021) and eye-hand coordination (e.g., Crippa et al., 2013).

Several studies show that active perception processes are compromised in ASD, which could lead to abnormalities in planning processes, serial, and prospective coordination (von Hofsten and Rosander, 2012). An ineffective information processing of the outside world may result in cognitive, language and social interaction impairments. Thus, by properly tracing the way of moving of ASD people within the environment, it might be possible to identify specific motor patterns that could help clinicians identify the disorder’s presence.

In this scenario, considering the need for a more reliable and timely diagnostic process for ASD, motion features could represent an effective precursor. Recent studies have begun to explore the predictive role of motion patterns as potential objective measures of the disorder, aiming at identifying a well-defined phenotype and enabling a computer-aided diagnosis perspective. These studies typically implement machine learning (ML) classification methods to predict or classify individuals of different groups by maximising the distance between groups of data sets. Several recent studies bolstered this argument showing that motor abnormalities could be a consistent marker of ASD and ML systems should be the method of choice to analyse them (Hyde et al., 2019). In particular, the potential of identifying such invisible and objective measures of ASD may enable early diagnosis of the disorder.

Recent studies in the field typically implement pattern classification methods based on supervised ML algorithms to predict or classify individuals of different groups by maximising the distance between groups of datasets. ML commonly refers to all procedures that train a computer algorithm to identify a complex pattern of data (i.e., “features”) that can then be used to predict group membership of new subjects. A supervised classification model learns rules from examples in different groups and uses these rules to predict unseen cases into perspective classes as accurately as possible. The model is first trained using labelled samples. On the other hand, unsupervised ML uses training data that do not include output information (i.e., labels) and can provide descriptive knowledge to help understand the data’s inherent structure or properties. Such ability to learn autonomously may represent the main potential benefit of artificial intelligence (AI) systems in supporting classical clinical diagnostic methods. Nevertheless, pattern classification methods can also identify complex patterns of anomalies not efficiently recognised by other statistical techniques. Thus, AI systems should not be considered merely from a potentially “diagnostic” perspective but also as a useful tool to develop objective measures of the disorder.

In this study, we deepened the analysis of a previous dataset (Simeoli et al., 2020, 2021) by applying a variational autoencoder, i.e., a particular type of artificial neural network. Such VAE is used to explore different prediction methods and broaden the ASD motion pattern analysis through latent distribution analysis. Specifically, VAE is an ML system that has the potential of “obtaining a joint distribution over all input variables through learning a generative model, which simulates how the data is generated in the real world” (Kingma and Welling, 2019; Huang and Zhang, 2022). VAE differs from traditional autoencoders by imposing restrictions on the distribution of latent variables, which allows it to find independent latent variables (Kingma and Welling, 2014). Following paragraphs describe it in detail.

## 2. Methods

This section introduces artificial neural networks (ANN) and variational autoencoders (VAE). Then, we describe the materials and methods used to obtain the datasets, how the data are preprocessed and finally, the network architecture and the hyperparameters.

### 2.1. Artificial neural network

**Figure 1:**
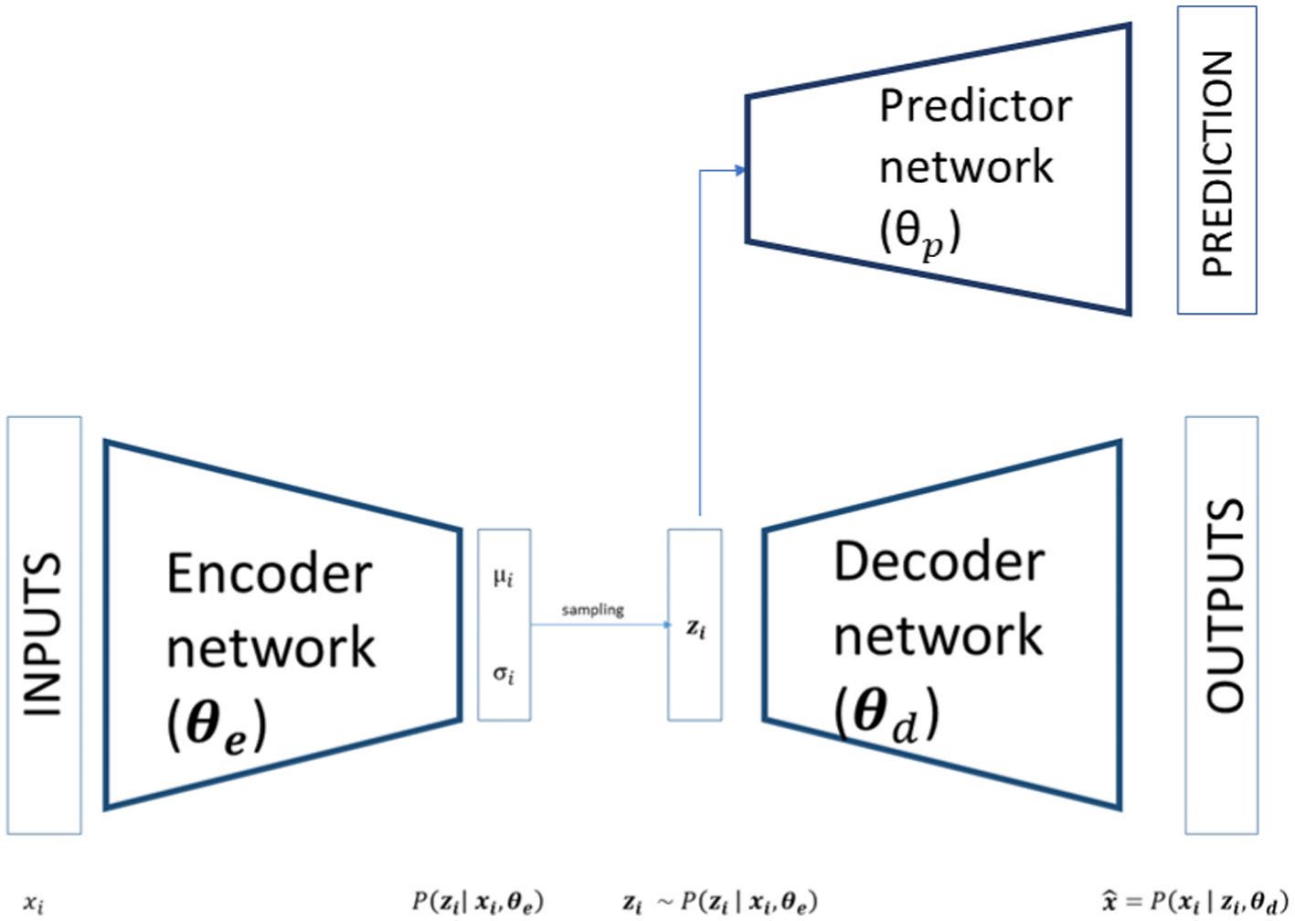
Schematic representation of a variational autoencoder with predictor network.

In its basic form, an ANN consists of an input layer of neurons (or nodes), one or two (or even more) hidden layers of neurons, and a final layer of output neurons: i.e. looking at Figure 1, the encoding, decoding and bottleneck layers of the depicted autoencoder are hidden layers, while the output layer is also the final layer. In Figure 1, connections between neurons are also shown. In the ANN each connection is associated with a numerical value called the connection’s weight (*W*). A neural network of this form is also called a feed-forward network because the signal passes layer by layer from the inputs to the outputs. The activation value of a hidden neuron is given by the weighted sum of all neurons from the previous layer, formally:


(1) $$h_{i}=\sigma \left(\sum_{j=1}^{N}W_{ij}x_{j}+b_{i}\right)$$

Where σ is the activation function of the neuron, *N* is the number of input neurons, $W_{ij}$ is the weight of the connection, and $b_{i}$ is a threshold term associated with the neurons called the bias.

A common example of an activation function is the sigmoid (or logistic) function, but several others have been introduced over the years.

It has been proved that, in principle, a neural network can learn to approximate any computable function to arbitrary precision. The variety of inputs a neural network can take, which goes from binary to real values or even symbols, and the capacity to mix different kinds of data as inputs without losing generality confer a wide range of applicability to ANNs. To learn to approximate a function, they need to adapt their weights to minimise the error between the output of the final layer and the actual outcome associated with a given input configuration. To this end, the backpropagation algorithm (Rumelhart et al., 1986) is used in layered feed-forward ANNs. The backpropagation algorithm uses supervised learning. That is, the experimenter provides the algorithm with examples of the inputs and expected outputs (the training dataset) she wants the network to compute. Those examples are used to calculate the error (i.e., the difference between actual and expected results). The idea of the backpropagation algorithm is to reduce the error until the ANN learns the training data. The training begins with random values for the connection weights, and the goal is to adjust them to minimise the error. The number of hidden layers and the number of neurons in each hidden layer must be fixed based on the application task, the complexity of the problem, and the number of inputs and outputs. Using a non-linear log-sigmoid activation function enables the network to simulate non-linearity in practical systems. Due to these numerous advantages and the number of variants and improvements to the original algorithm proposed over the years, backpropagation is the most used and known algorithm to train neural networks.

### 2.2. Variational autoencoder

VAE is an ANN model specifically designed to learn interpretable generative factors, typically non-linear, throughout neural networks and backpropagation training (Rumelhart et al., 1986; Kingma and Welling, 2014). It comprises two symmetrical neural networks, an encoder and a decoder, connected by a hidden layer that maps the inputs in a low-dimensional space, usually referred to as latent space. The encoder part of a VAE models the distribution $q\left(z_{i}\right)=P\left(z_{i}\;|\;x_{i},\theta_{e}\right),$ where $x_{i}$ are the inputs, $\theta_{e}$ the weights of the VAE’s encoder and $z_{i}$ the latent variables. As showed in Figure 1, the outputs of the encoder are the parameters of a distribution. Theoretically any distribution can be chosen but usually a multivariate Gaussian distribution is used most of the time. If the choice is restricted to a diagonal Gaussian distribution we have only two sets of output neurons for each latent variables, referring to the mean $\left(\mu_{i}\right)$ and to the standard deviation $\left(\sigma_{i}\right)$ of the latent variables. In this way the inputs are mapped to a low dimensional space that has a well-defined distribution from where we can sample our latent variables:


(2) $$z_{i}∼q\left(z_{i}\right)=P\left(z_{i}\;|\;x_{i},\theta_{e}\right)$$

As shown in Figure 1 this latent variable is then passed to the decoder part of the network that tries to reconstruct the input starting from a compact latent representation. The structure of the decoder is symmetrical respect to the encoder and models a conditional distribution $P\left(x_{i}\;|\;z_{i},\theta_{d}\right)$ where $\theta_{d}$ are the weights of the decoder. The outputs of the decoder, $\hat{x}$, can directly compute the reconstructed data or, as in the encoder case, determine the parameters of the conditional distribution.

In VAEs the loss function is composed of a reconstruction term, usually classical loss functions like mean squared error or binary cross-entropy, and a regularisation term that ensures the regularity of the latent space and the correct approximation to our chosen conditional distribution. This regularisation term is expressed as the Kulback–Leibler divergence between the returned latent distribution and, in our case, a diagonal gaussian distribution with 0 mean and standard deviation 1.


(3) $$\text{lossVAE}=L_{\text{recon}}+KL\left[q\left(z_{i}\right),N\left(0,1\right)\right]$$

Where $L_{\text{recon}}$ is a classical reconstruction error of the data, in this case we used the mean squared error, and $KL\left[q\left(z_{i}\right),N\left(0,1\right)\right]$ is the Kulback–Leibler divergence between our latent distribution and a diagonal gaussian distribution with zero mean and unitary variance.

For this study, we added to the VAE a predictor network that connects the latent space to an additional predictive output. This network is a simple multilayer perceptron, with wheights $\theta_{p}$, that takes as input the latent variables $z_{i}$, process the latent variables through an intermediate layer with a *tanh* activation function and then classifies the subject based on a *softmax* activation function. In our case, we have two classes, so we use a binary cross-entropy for the predictor loss function:


(4) $$L_{\text{pred}}=-\left(y\log\left(p\right)+\left(1-y\right)\log\left(1-p\right)\right)$$

Where *y* is a binary indicator returning the true label of a subject and p is the predicted probability for a given observation to belong to that class.

The whole loss function of our model is given by three terms: the reconstruction error of the autoencoder; the regularisation of the latent space; and the prediction error of the predictor network. Formally:


(5) $$\text{loss}=L_{\text{pred}}+L_{\text{recon}}+KL\left[q\left(z_{i}\right),N\left(0,1\right)\right]$$

Where all the components of the loss are equally weighted.

### 2.3. Participants

The study was attended by 60 children aged between 5 and 10 years. The sample was arranged into autism spectrum disorder (ASD) and typically developing (TD) groups. The ASD group includes 30 children with an average age of 7 years ± 1.4, clinically diagnosed with ASD according to the Diagnostic and Statistical Manual of Mental Disorders (5th Ed.). Inclusion criteria were as follows: (i) diagnosis of ASD according to both DSM-V clinical criteria and to the Autism Diagnostic Observation Schedule (ADOS-2) (Lord et al., 2012), (ii) age range between 5 and 10 years, and (iii) no existing comorbidities.

All participants in the ASD group were diagnosed with ASD by qualified doctors and professionals in the sector.

The TD group includes 30 children, with a mean age of 6 years and 8 months ± 1, without any confirmed neurodevelopmental disorder. Exclusion criteria were: (i) suspected signs of ASD, (ii) developmental abnormalities, and (iii) current or past history of psychiatric or neurological disorders.

All participants had normal vision and no sensory or motor deficits. Any child whose clinician or teacher was uncertain about the child’s diagnosis or health was excluded.

IQ was assessed for the entire sample using the Leiter-3 International Performance Scale. The IQ score for the TD group ranged between 74 and 110, while the ASD group covered a range from 59 to 109. Six children in the ASD group had mild mental retardation with an IQ score between 59 and 70 (World Health Organisation [WHO], 2016). No severe or profound mental retardation was detected.

The ASD participants were recruited from the Neapolisanit S.R.L. Rehabilitation Center. The TD participants were recruited from a primary school.

Children with ASD followed psychomotor and speech therapy treatment at the Neapolisanit S.R.L. Center.

### 2.4. Materials

The motion detection software was developed in Unity and C# using an Android tablet 6.0, size (H × W × D) 241.9 × 149.5 × 8.5 mm, screen size 9.6 inches with a resolution of 1,280 × 800 (WXGA) and a refresh rate of 60 Hz. The task consists of a sequence of scenes which play cognitive tasks from the Leiter-3 test Cognitive Battery (Roid et al., 2013). Participants were asked to perform the tasks following the same original test procedure. The examiner switched from one subtest to the other according to the instruction procedure of the original test version.

Scenes comprise a maximum of five moving cards and eight fixed images (placeholders). Moving cards are generally placed at the bottom of the screen and can be dragged, and placeholders are placed at the top. Placeholders are designed to catch the moving cards when they are dragged nearby.

The software performs five subtasks, each investigating different cognitive domains. Tasks require the user to drag the moving cards from the bottom of the screen to the placeholders at the top and provide a progressive increase of distracting stimuli and details of the images.

For this study, we only analysed the trajectories drawn during the tasks correctly performed to avoid the noise of the “cognitive” mistakes for the classification process.

During the task, participants sat in front of a table 65–70 cm high, the experimenter sat on the opposite side. Children performed the task on the tablet placed within 20 cm of the table’s edge. At the beginning of each subtest, the examiner provided instructions according to the instruction procedures of the original version of the test, including a series of guides that encouraged attention to the main cognitive target, using pointing and specific gesture guides, without any vocal aid. After the instructions, the examiner left the child free to perform the task without further aid.

The task was considered complete when each of the moving cards was placed into one of the placeholders at the top, regardless of the correctness of the answer.

### 2.5. Features extraction

The software recorded information about the participant’s finger position during the dragging task, namely 40 pair coordinates (*x*, *y*) of movement per second were collected runtime at a rate of 40 Hz.

We considered as “trajectory” the set of coordinates resulting from the first tap on the screen until the finger lifted at the end of each dragging movement. For each “trajectory” we obtained the specific value for each of the 12 features described in Table 1. The analysis was conducted, and features were extracted for each trajectory. Features were extracted from sets of raw coordinates using RStudio software and the *traj* package (Leffondree et al., 2004; Sylvestre et al., 2006).

**Table 1:** Features description.

Feature	Description
1. MeanSpeed	Average speed values per task
2. MaxSpeed	Average value of the maximum speed peaks reached during the performance of a task
3. MinSpeed	Average value of the minimum speed peaks reached during the performance of a task
4. sdSpeed	Standard deviation of the speed values collected during the task
5. MeanAcceleration	Average acceleration values per task
6. MaxAcceleration	Average value of the maximum acceleration peaks reached during the performance of a task
7. MinAcceleration	Average value of the minimum acceleration peaks reached during the performance of a task
8. sdAcceleration	Standard deviation of the mean acceleration values collected during the task
9. STH	The ratio between the distance of the starting and ending points of a trajectory and its length
10. DC	The change in direction over time
11. sdDC	Standard deviation of directional change value obtained during the task
12. MeanLength	The average amount of finite trajectories conducted during each task

STH: straightness is a number ranging from 0 to 1, where 1 indicates a straight line. It is an index of linearity. DC: directional Change is defined for each pair of steps so that a trajectory may be characterised by the mean (DC) and standard deviation (sdDC) of all directional changes. DC may be used as an index of nonlinearity, and sdDC as a measure of irregularity.

For the analysis, we used the average value merged per task. These features provide a comprehensive computational description of the child’s motion patterns. Data description concerned: (i) kinematics information, e.g., speed or acceleration and (ii) touch-based functions, e.g., the number of trajectories drawn during the task and the average length (Table 1).

All these data were then aggregated to find the average values for each task. The final dataset consisted of the mean value for all the features (Table 1) divided per task (five difficulty levels). The participants performed 25 tasks, 5 for each difficulty level, so our final dataset was composed by 1,500 × 12 observations. The target dataset is instead a simple vector of 1500 × 1 labels where the diagnosis of the participants is reported, 0 for TD participants and 1 for ASD.

## 3. Results

In order to analyse the features’ impact on classification and provide useful additional measures to clinicians for ASD detection, we used the VAE to generate meaningful latent space to disentangle the predictor response and analyse the impact of each feature on the latent mapping.

The following sections report the training result of the complete model and the results of features’ impact on classification, obtained discarding predictor and using the learned distribution of the latent space.

### 3.1. Prediction results and latent space distribution

To verify the model’s classification accuracy, we trained the model on the collected dataset. Each observation is passed as input to the model that predicts the participant’s diagnosis considering that specific task. At the same time, the model tries to reconstruct the input creating a meaningful latent space as described in the method section. To prevent overfitting, we used the k-fold cross-validation with *k* = 10 and returning the average accuracy. We trained a model with 10 latent variables and applied a Principal Component Analysis on the latent space to choose the right number of latent variables defining the latent space. We found that the first two latent variables explain the 95% of the variance of the latent space; so, for the final and subsequent analysis, we restrict our choice to two latent variables.

After the *k*-fold cross-validation, the average accuracy reached the 91.2% ± 5.6% on the test set.

In order to see how the subjects are mapped in the latent space we plot the mean $\mu_{i}$ of the latent distribution $z_{i}$ learned by the VAE for each subject *i*, since the mean is usually a good representation of the latent projection of a subject (Figure 1). To this end, we choose one of the trained models from the previous *k*-fold training. We found that the latent mapping is independent of the weights initialisation, and the learned structure of the latent space is consistent across different trainings by taking into account a rotation factor, as already found in other works (Huang and Zhang, 2022).

**Figure 2:**
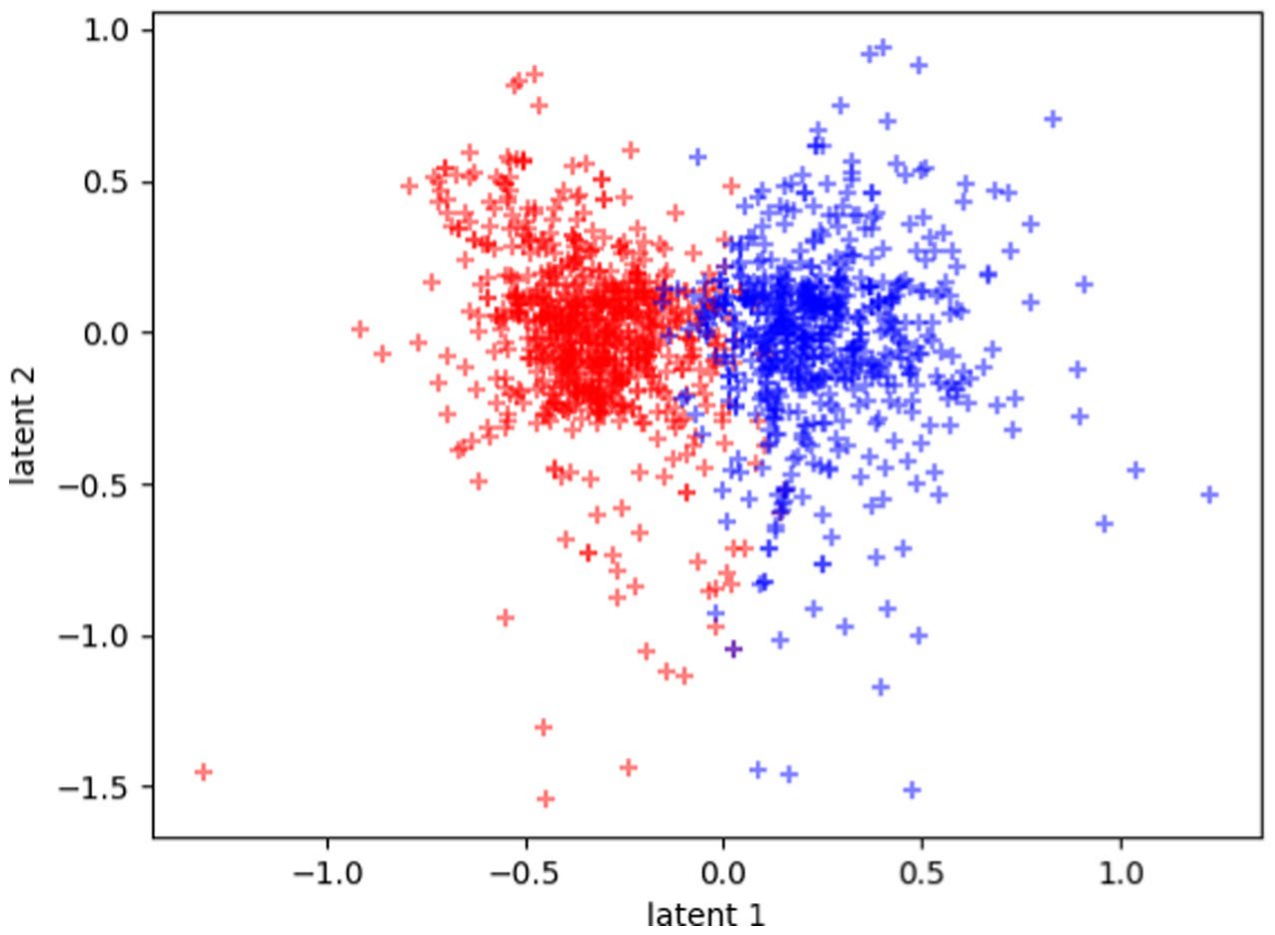
Plot of the latent space learned by the model. Blue dots represent the center values of tasks belonging to ASD group, red dots represent TD’s ones.

As we can see in Figure 2, which shows the latent distribution of the tasks belonging to the two groups (ASD, TD), the latent space is automatically clustered, namely, data from the tasks of the two groups are mapped in different zones of the latent space and describes two different distributions. The latent space regularisation imposed by the VAE loss function provides this automated clustering. This happens because the latent space sampling is passed to the predictor network. Indeed, if the latent distributions of the two classes are too noisy and overlapped, the prediction loss will quickly stagnate in a local minimum. Finding a separated distributional form for the two classes in the latent space ensures that the stochastic sampling process from the latent space is not noisy among the two classes and the predictor can learn to classify well.

**Figure 3:**
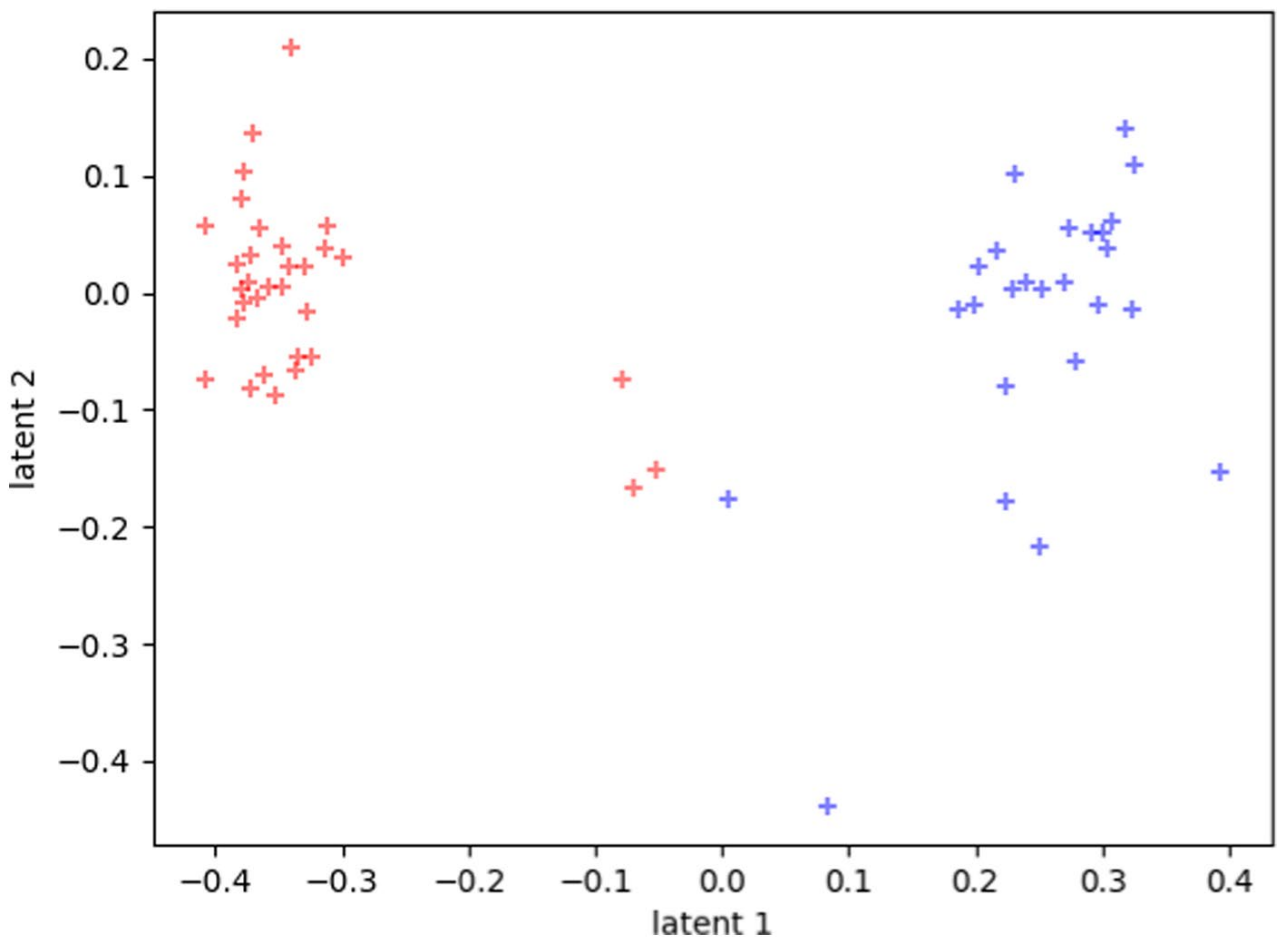
Latent representations of the 60 participants. Each dot represents the average value of all the tasks for each participant. Red dots represent TD distribution in the space, and blue dots represent ASD distribution in the space.

The clustering is even more evident if we plot each participant in the latent space as the mean of all their tasks, rather than each single task separately, as reported in Figure 3.

### 3.2. Features analysis of the latent space

At this point, we have retrieved a meaningful representation of the latent space, which correctly separates the participants in two classes. To better understand the role of the features in the classification process, we analyse which input features are capable of changing the position of a participant in the latent space and the probability of belonging to one of the two distributions. To this end, we fit the two learned distributions over the latent variables using a multivariate bi-dimensional gaussian diagonal distribution instead of the predictor network. Specifically, we used this function for the fitting because the regularisation loss of the latent space imposes this same structure on the latent space. Doing this, we retrieve the functional form of the two distributions and measure the probability density function for a point in the latent space to be assigned to one of the two classes. Moreover, we used the fitted distribution instead of the predictor network, to prevent the predictor from incorrect classification, i.e., new participants being mapped in unknown zones of the latent space. Indeed, restricting the analysis to the latent distributions reduces the probability of misclassifying new inputs. Using latent metrics to make inferences and predictions is common in outlier detection experiments (Zhou and Paffenroth, 2017). Here we use the latent space instead of the predictor network to avoid that new inputs could be misclassified, that is, imposing a more restrictive metric that prevents incorrect diagnosis of unseen inputs.

To measure how each feature impacts the latent space mapping of a participant, we perform a Monte Carlo sampling of each feature while keeping the others fixed. More formally, if we call **x** an input vector composed of our twelve features:


(6) $$x=\left(f_{1},f_{2},\ldots \ldots ,f_{12}\right)$$

We modify **x** sampling the features *i* as:


(7) $$f_{i}∼N\left(\mu_{f_{i}},\sigma_{f_{i}}\right),i=1:12$$

While the other features are unchanged. By doing 100 random samples for each feature, we can measure how the given feature variation modifies an input’s position in the latent space.

**Figure 4:**
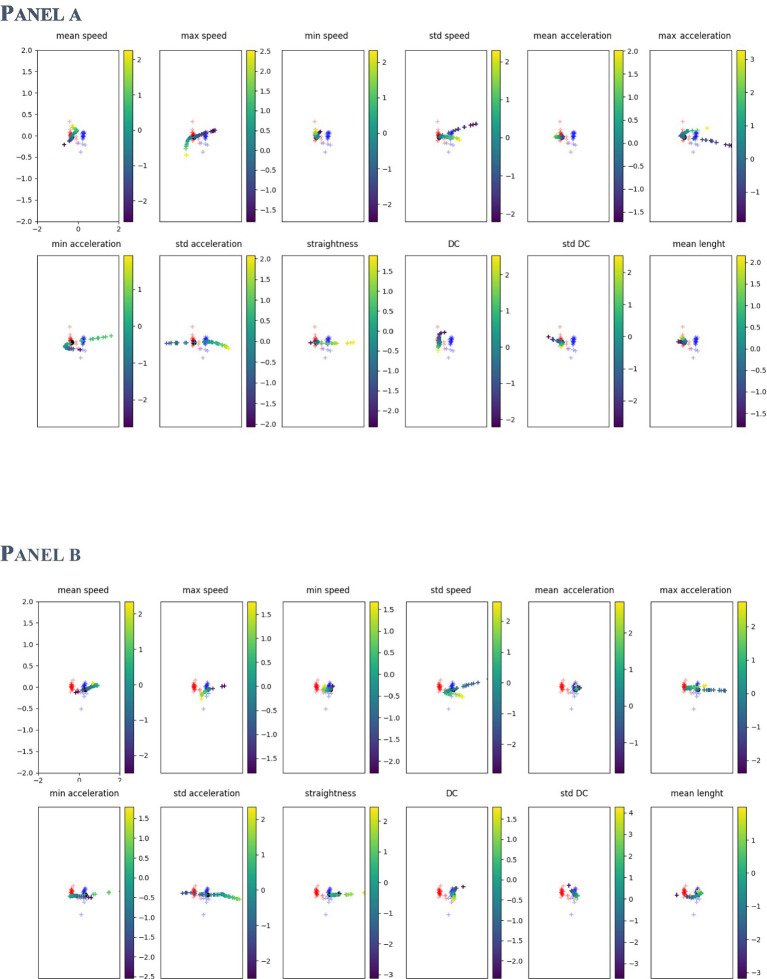
Trajectories in the latent space of two participants (Panel **(A)**: TD; Panel **(B)**: ASD) varying the test features. Latent distribution of the two classes (red and blue dots) are reported for clarity. Each subfigure shows the different positions of the participant in the latent space when that specific feature value is modified. The dot’s color change as the feature value changes.

In Figure 4 two examples are reported, one for a TD subject (Panel (A)) and one for a ASD one (Panel (B)). Starting from the subject projection in the latent space, we modified all his tests, one feature at the time, and plotted the new mean position in the latent space. Results revealed that some features determine a different encoding in the latent space, moving the subject far away from its original position. For some specific values of those features, the classification of the input is reversed, matching the opposite distribution to the one he belongs to.

**Figure 5:**
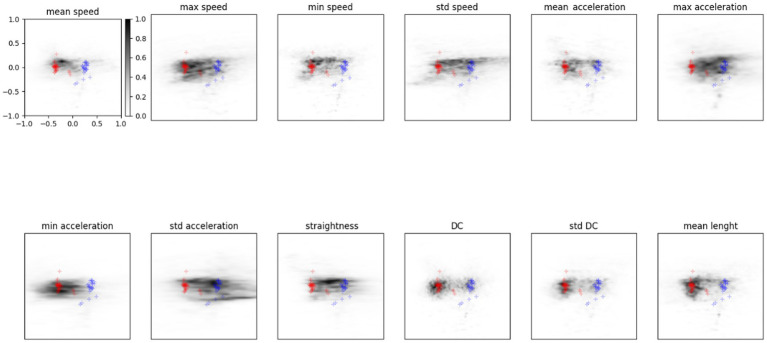
Probability density in the latent space for each feature. Red points show the TD distribution described before, blue represents the ASD one. The grey scale indicates the probability for a feature to be mapped in that zone of the latent space.

Figure 5 shows the probability for a feature to be mapped in a particular zone of the latent space. Results refer to the average of the analysis described by Equation (7) computed on the entire dataset of participants. The grey scale indicates the density of points in the latent space covered by a feature variation: The darker the area, the higher the probability of being mapped there. The features that visually appear to spread more between the two distributions are sdSpeed, MaxAcceleration, MinAcceleration, and sdAcceleration. While other features, like sdDC, MeanSpeed, MaxSpeed, and MeanLenght, are mapped preferentially towards zones of the latent space covered by the TD group distribution. We assumed that these latter features could be marginal for ASD classification.

### 3.3. Features’ impact on latent classification

For a more quantitative analysis, we measure the probability of a point in the latent space being assigned to one of the two latent distributions. We use the probability density function of the gaussian distributions derived from fitting the points in the latent space. In this way, we can measure how a variation in the features can affect a subject’s latent projection and his consequent correct classification.

**Figure 6:**
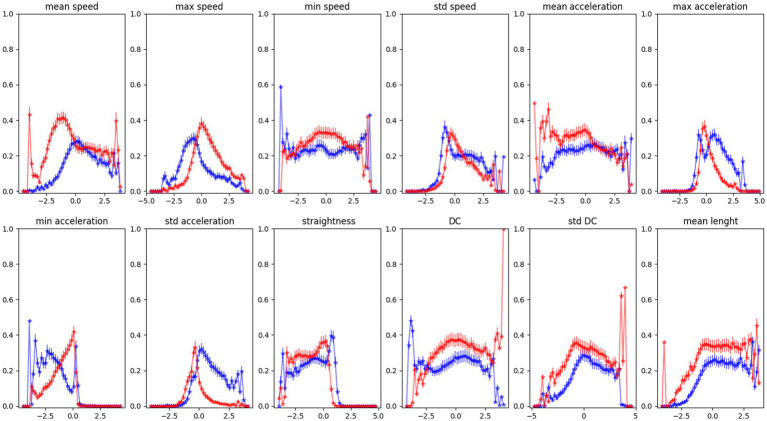
Red and blue curves are, respectively, the probability to be assigned to TD and ASD distribution in the latent space with different feature values. Data averaged over all subjects, mean and standard deviation for each point are reported.

Figure 6 reports each feature’s probability density function (PDF) against its values. We can see how the probability of belonging to one of our latent distributions varies across the feature values. For some features, the probability of being closer to the ASD latent distribution is higher for high feature values, as in the case of MaxAcceleration and sdAcceleration. On the contrary, low feature values correspond to a higher probability of belonging to ASD distribution for MaxSpeed and MinAcceleration.

## 4. Discussion

In recent decades the clinical approach to assessing and diagnosing ASD has been based on measuring several explicit behavioural variables, assuming a qualitative value. For this reason, researchers have been trying to identify quantitative measures of the disorder in the last few years.

In this scenario, embodied cognition theory and predictive coding principle (Friston and Kiebel, 2009; De Jaegher, 2013) served as a theoretical framework for a new methodological approach focusing on sensory processing analysis and motor abnormalities and general body-world interaction as potential symptomatology indicators. Recent studies have raised the problem of categorising and recognising autism through these measures, suggesting the exploitation of the potential of the latest technological advances and artificial learning methods (Hyde et al., 2019).

This study fully fits into this framework, trying to delve into the topic and specifically aiming at identifying quantitative measures of the disorder and their respective threshold values.

Using ANN and VAE methods, we analysed kinematic features of simple dragging movements managing to classify ASD and TD individuals. The VAE methods describe the ASD and TD distributions (Figure 5) according to each feature value and its variation in the latent space.

In the first study (Simeoli et al., 2020), by using descriptive analysis, we observed some specificity of the ASD motion pattern. Results revealed that ASD trajectories were characterised by low linearity, high MaxSpeed and MaxAcceleration values, and low MinumumSpeed and MinimumAcceleration values. However, it was impossible to distinguish significant differences between the two groups. Conversely, in this study, we described the difference between the two distributions (ASD and TD). In particular, by modifying the value of the features within the latent space, as shown in Figures 4, 5, we observed the potential role of each feature for classification. The features that appear more effective for classifying ASD individuals are sdSpeed, MaxAcceleration, MinAcceleration and sdAcceleration. Conversely, sdDC, MeanSpeed, MaxSpeed, and MeanLenght are mapped preferentially within zones of the latent space covered by the TD distribution. Therefore, we assume these latter features could be marginal for the ASD classification. Furthermore, our results suggest that the probability of being classified in the ASD distribution depends on specific values of those features. Specifically, using the probability density functions derived by the VAE, we observed that higher values of MaxAcceleration and sdAcceleration, and lower values of MaxSpeed and MinAcceleration increase the probability of being classified as ASD. These measures could be used as threshold values by clinicians. Using a joint probability of these features, they could identify suspected clinical conditions and use these measures to enhance the assessment and diagnostic process.

One potential limitation of this study is the use of a classification method at the begging of the process. Indeed, because of the sample size and its characteristics, it was necessary to train the network according to the original labels of the two groups and then build the latent space and the respective distributions. Therefore, we had to rely on the traditional assessment method to provide the ground truth to the model. However, optimal VAE functioning could be entirely unsupervised, and this study moves that way.

Considering our findings, a future study will improve the dataset according to size and features relevance to obtain an unsupervised organisation of the latent space distributions. In general, future studies in this field are encouraged to generate as many features as possible to allow for the specification of the globally optimal set of features for ASD identification (Zhao et al., 2021). These methods should be empowered to determine which and how a variety of features could be identified and effectively used in clinical practice to obtain a more comprehensive multimodal measure of the risk of ASD and support the diagnostic process for early detection.

## 5. Conclusion

This study reveals that ML systems may benefit traditional diagnostic and assessment methods significantly. However, performance can be improved by tailoring future datasets for the exact usage of the systems. The proposed method provides important implications for ASD classification and its phenotypical descriptions. It may open novel avenues for clinical screening and provide a potentially accurate ML architecture for researchers and clinicians to analyse new data. Future research should aim to collect more clinical data to cover more latent space and obtain a trustworthy decision-support system to predict new data. Moreover, these quantitative measures can be combined with the traditional behavioural and cognitive scale, further supporting clinicians in their clinical process.

## Data availability statement

The raw data supporting the conclusions of this article will be made available by the authors, without undue reservation.

## Ethics statement

The studies involving human participants were reviewed and approved by Ethical Committee of Psychological Research of the Department of Humanities of the University of Naples Federico II. Written informed consent to participate in this study was provided by the participants’ legal guardian/next of kin.

## Author contributions

RS contributed to the conception and design of the study, developed the software and carried out the experimental sessions, contributed to the data analysis, and drafted the manuscript. NM contributed to the data analysis developing the machine learning model and drafted the manuscript. AR provided substantial contributions to the acquisition of data and coordinating the experimental work. DM supervised the work, contributed to the conception and design of the study, and coordinated the data analysis and software development process. All authors contributed to the article and approved the submitted version.

## Conflict of interest

RS and AR were employed by Neapolisanit S.R.L. Rehabilitation Center.

The remaining authors declare that the research was conducted in the absence of any commercial or financial relationships that could be construed as a potential conflict of interest.

## Publisher’s note

All claims expressed in this article are solely those of the authors and do not necessarily represent those of their affiliated organizations, or those of the publisher, the editors and the reviewers. Any product that may be evaluated in this article, or claim that may be made by its manufacturer, is not guaranteed or endorsed by the publisher.
